# Genomic features of Chinese small cell lung cancer

**DOI:** 10.1186/s12920-022-01255-3

**Published:** 2022-05-20

**Authors:** Jun Liu, Zhuxiang Zhao, Shuquan Wei, Binkai Li, Ziwen Zhao

**Affiliations:** Department of Pulmonary and Critical Care Medicine, The Second Affiliated Hospital of South China University of Technology, Guangzhou, 510000 China

**Keywords:** Small cell lung cancer, Germline, DNA damage repair, TMB, Actionable alterations

## Abstract

**Background:**

Small cell lung cancer (SCLC) is an aggressive disease with poor survival. Although molecular and clinical characteristics have been established for SCLC in western patients, limited investigation has been performed for Chinese SCLC patients.

**Objective:**

In this study, we investigated the genomic features of Chinese SCLC patients.

**Methods:**

A total of 75 SCLC patients were enrolled. Genomic alterations in 618 selected genes were analyzed by targeted next-generation sequencing.

**Results:**

Here, we showed that *TP53* (77.30%) and *RB1* (30.70%) were the most prevalent genes alterations, followed by *KMT2D, ALK, LRP1B, EGFR, NOTCH3, AR, CREBBP, ROS1,* and *BRCA2*. And the most common genetic alterations were enriched in the cell cycle signaling pathway (84.00%) of Chinese SCLC patients. DNA damage repair (DDR) pathway analysis showed that the most frequently enriched DDR pathways were fanconi anaemia (FA, 29.41%) and homology recombination (HR, 21.57%). Notably, 9.33% SCLC patients in our cohort had pathogenic or likely pathogenic germline gene variants. Compared with the U Cologne cohort, a higher prevalence in *EGFR, AR, BRCA2, TSC1, ATXN3, MET, MSH2, ERBB3* and *FOXA1* were found in our cohort; while compared to the data from the Johns Hopkins cohort, a higher mutated frequency in *TP53, KMT2D, ALK*, and *EGFR* were found in our cohort. Moreover, a significant association was found between high tumor mutation burden (TMB) and mutations involved in *TP53*, *CREBBP, EPHA3, KMT2D, ALK* and *RB1*. Approximately 33.33% of patients with SCLC harbored at least one actionable alteration annotated by OncoKB, of which one patient had alterations of level 1; seventeen patients had level 3; fifteen patients possessed level 4.

**Conclusion:**

Our data might provide an insightful meaning in targeted therapy for Chinese SCLC patients.

**Supplementary Information:**

The online version contains supplementary material available at 10.1186/s12920-022-01255-3.

## Introduction

Small cell lung cancer (SCLC) is a highly malignant form of lung cancer that kills ~ 250,000 people worldwide annually and accounts for approximately 15% of lung cancer cases [1]. Biologically, rapid doubling time and early widespread metastases are characteristic of SCLC. Around 70% of cases present with the extensive-stage disease at diagnosis (ES-SCLC); the remaining 30% of patients have the limited-stage disease (LS-SCLC), in which tumor involvement is confined to one hemithorax and can be treated in a tolerable radiation field. The overall prognosis of SCLC patients is poor, with a median overall survival (OS) of 15–20 months for LS-SCLC and 8–13 months for ES-SCLC [2, 3].

Chemotherapy has been the bedrock of the treatment of SCLC for over two decades, now is replaced by immuno-chemotherapy strategy. Compared with chemotherapy alone, combination therapy with atezolizumab, an anti-program death ligand 1 (PD-L1) antibody and chemotherapy as the first-line treatment of ES-SCLC significantly prolonged overall and progression-free survival [4]. In a subsequent study, durvalumab, another PD-L1 antibody, in combination with platinum and etoposide also significantly improved overall survival in ES-SCLC patients [5]. Although the advent of immunotherapy has benefited SCLC patients, with only a modest efficacy, compared to other solid tumors. In addition, there is still a lack of targeted therapy for SCLC. Therefore, therapeutic strategy for SCLC treatment still has a lot of room for improve, and there are many problems and limitations that need to be solved urgently.

Some studies based on Caucasian population identified alterations in *TP53* and *RB1* were the most prevalent in SCLC [6–8]. In addition, *PIK3CA*, *EGFR* and *KRAS* also have high mutation frequency in SCLC [6]. Specifically, biallelic inactivation of *TP53* and *RB1* can be detected in almost all the SCLC tumors, suggesting that loss of the tumor suppressors *TP53* and *RB1* is obligatory in SCLC [6]. However, mutations in other genes varied from study to study. The majority of mutations have little significance for the SCLC pathogenesis and are described as passenger mutations. Finding the driving mutations of heterogenous diseases among SCLC patients and developing them into actionable targets for treatment are the primary issues to be faced [9]. There are very few genomic data of SCLC in China. In order to fill the gap of comprehensive genomic variation of SCLC, it is necessary to track more genomic variation of SCLC from different populations. In addition, the prognostic value of mutated genes in SCLC has not been well investigated.

With the in-depth research on the mechanism of DNA damage repair (DDR), people have a further understanding of improving sensitivity and overcoming resistance to traditional DNA damage treatment [10]. Although DDR data are scarce in SCLC, Byers et al. identified the DNA repair protein poly ADP-ribose polymerase 1 (PARP 1) as a therapeutic target [11]. Preclinical SCLC models were sensitive to PARP inhibition alone and the efficacy of chemotherapy was also enhanced by the addition of a PARP inhibitor [12, 13]. Despite of this, definite recurrent and targetable genomic alterations have not been identified in SCLC at present, especially in the Chinese population. Moreover, the DDR profile of Chinese SCLC patients was still not very clear yet.

Here, we carried out this study to clarify the genomic alterations and molecular characteristics of Chinese SCLC patients, especially DDR alterations and TMB levels. We attempted to better understand the association of genomic alterations with TMB levels in SCLC, and identify candidate prognostic biomarkers. Additionally, we tried to figure out whether there were significant differences in the mutational data between our cohort and the other two cohorts from cBioportal database. We further investigated the germline mutations and defined the frequency of actionable alterations to catch sight of the genetic features as well as corresponding target therapies in Chinese SCLC patients.

## Materials and methods

### Biospecimen collection and clinical data

Biospecimens of 75 SCLC patients were collected. All patients provided written informed consent for publication of their clinical details. Formalin-fixed, paraffin-embedded (FFPE) tumor tissues were pathologically assessed to have at least 20% tumor cells. Blood samples were drawn into Cell-free DNA BCT tubes (Streck, Inc.). Blood Cell-free DNA (cfDNA) testing were performed in 50 patients who could not provide sufficient or valid tumor tissue samples.

### DNA isolation

The FFPE samples and peripheral blood mononuclear cells were collected using DNeasy Blood &Tissue Kit (Qiagen, Inc.) to isolate gDNA following the manufacturer’s instruction [14]. cfDNA was extracted from blood was using the QIAamp Circulating Nucleic Acid Kit (Qiagen, Inc.) according to the protocol of the manufacturer. The purified gDNA and cfDNA were quantified using the Qubit 3.0 Fluorometer (Life Technologies, Inc.) and StepOnePlus System (Life Technologies, Inc.) [14].

### Target next-generation sequencing

For the tumor and blood samples, 100 ng gDNA was sheared to target 200 bp fragment sizes with the Covaris E210 system (Covaris, Inc.). Next-generation sequencing of gDNA and cfDNA was performed, in which Accel-NGS 2S DNA Library Kit (Swift Biosciences, Inc.) was used for library preparation and xGen Lockdown Probes kit (IDT, Inc.) for target enrichment [14]. The custom xGen Lockdown probe was synthesized by IDT, Inc. for the exons and selected intronic regions of 618 genes (Additional file 1: Table S1). The prepared library was quantified using the Qubit 3.0 Fluorometer (Life Technologies, Inc.), and quality and fragment size were measured with an Agilent 2100 Bioanalyzer (Agilent Technologies, Inc.). Samples underwent paired-end sequencing on an Illumina Nextseq CN500 platform (Illumina Inc) with a 150-bp read length [15]. The mean coverage of tumor gDNA, blood cfDNA and peripheral blood mononuclear cells was more than 1000 × , 3500 × and 200 × , respectively.

### Tumor mutation burden analysis

Tumor mutation burden (TMB) was defined as the total somatic nonsynonymous mutation counts in coding regions [16]. TMB was classified into high and low categories, with the top quartile as the cutoff value.

### Interpretation of pathogenicity of germline variants

Variants were detected in the white blood cells with at least 8 supporting reads and allele frequency beyond 20% were considered as germline variants. Then those variants with population allele frequency over 1% (from 1000 genomes and ExAC database), labeled as benign or likely benign in the latest Clinvar database and/or synonymous were excluded. The interpretation of germline variants followed the standards and guidelines of American College of Medical Genetics and Genomics and the Association for Molecular Pathology (ACMG/AMP) and independently reviewed by two genetic consultants [17].

### Data and statistical analysis

Raw sequencing data were aligned to the reference human genome (UCSC hg19) through Burrows-Wheeler Aligner and producing a BAM (binary alignment/map) file [18]. After removing duplicate and local realignment, single nucleotide variation (SNV)/indel calls were performed using the Genome Analysis Toolkit (GATK) [19]. Somatic variants were generated for the patient by subtracting the germline variants from the tumor to keep only variants unique to a tumor. Variants were annotated using the ANNOVAR software tool. Somatic mutations were annotated with information from the Catalog of Somatic Mutations in Cancer (COSMIC) database [20]. The Genomic alterations data of Johns Hopkins, Nat Genet 2012 (80 patients) and U Cologne Nature 2015 (120 patients) was downloaded from OncoKB (https://www.oncokb.org/) [21]. The survival data was downloaded from National Center for Biotechnology Information (NCBI, https://www.ncbi.nlm.nih.gov/pmc/). Differential mutations analysis was performed under a dominant model using Chi Square test or Fisher exact test. P values less than 0.05 on two-sides were considered statistically significant. All analyses were performed by SPSS 25.0 software.

## Results

### Clinicopathological characteristics of SCLC patients

This study enrolled a total of 75 Chinese SCLC patients, among whom 52 were males and 23 patients were female. The clinical characteristic obtained are summarized in Table 1. The ages of the patients ranged from 39 to 89 with a median age of 66. Eight (10.7%) SCLC patients had been diagnosed with II-III stage, and 67 (89.3%) patients with IV stage. Moreover, 18 (24.0%) cases presented a family cancer history, and 55 (73.3%) individuals without it. All tumor samples were pathologically assessed to have a purity of at least 20%.

**Table 1:** Clinical Characteristics of 75 SCLC Patients

Clinicopathologic parameter		Chinese SCLC patients (N = 75)
Age	Median Age (Range)	66 (39–89)
Gender	Male	52 (69.3%)
	Female	23 (30.7%)
Sample Type	Blood	53 (70.7%)
	Tissue	22 (29.3%)
Pathology stage	II-III	8 (10.7%)
	IV	67 (89.3%)
Family cancer history	Yes	18 (24.0%)
	No	55 (73.3%)
	Unknown	2 (2.7%)
Smoking status	Smoker	6 (8.0%)
	Non-Smoker	1 (1.3%)
	Unknown	68 (90.7%)

### The landscape of mutation profiles in SCLC

**Fig. 1:**
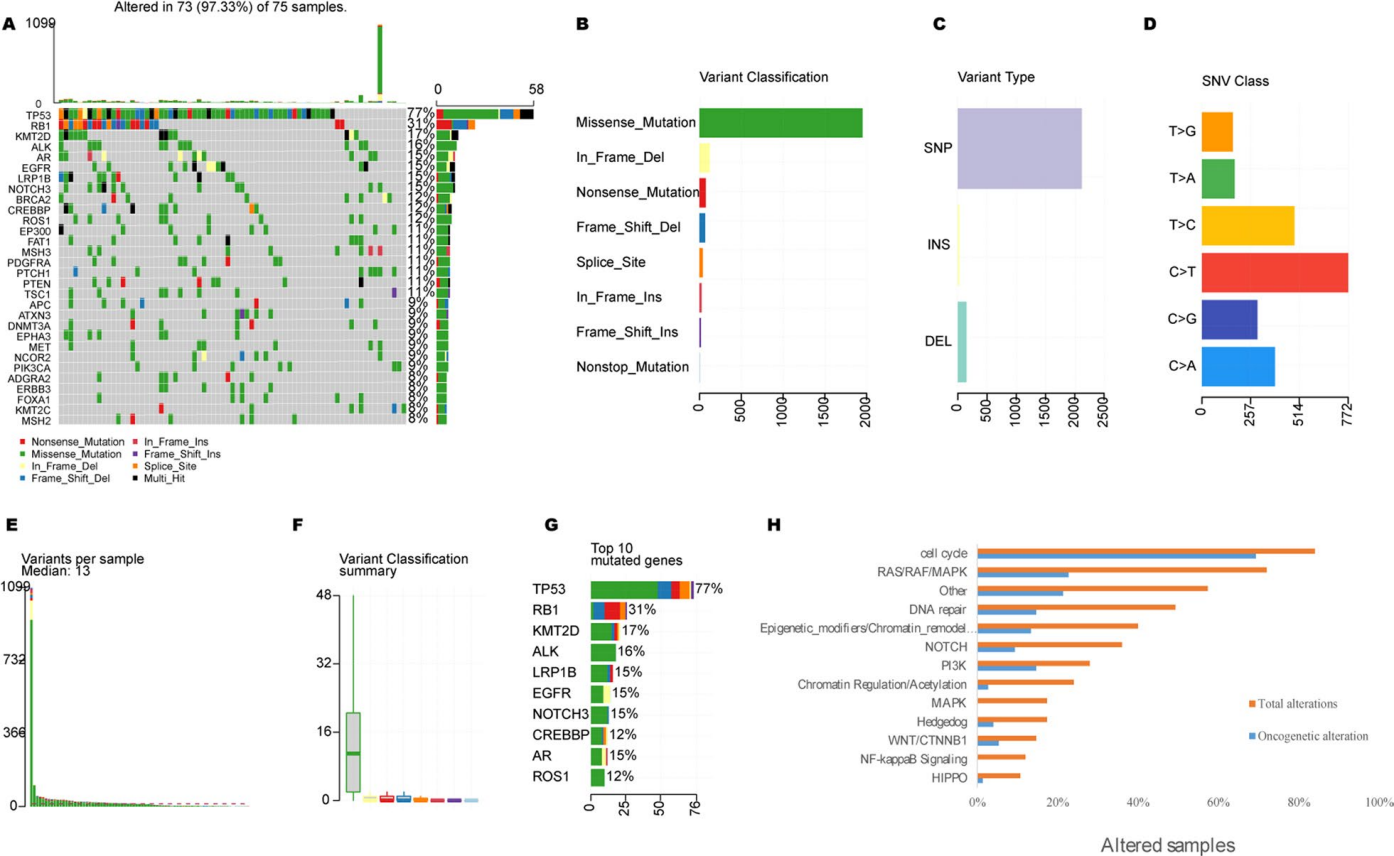
The landscape of mutated genes in a series of 75 SCLC patients. **A** Oncoprint of the 30 most frequently mutated genes in our cohort. **B** Summary of the mutation information with statistical calculations. **C**, **D** Classification of mutation types according to different categories, in which missense mutation accounts for the most fraction, SNP showed more frequency than insertion or deletion, and C > T was the most common of SNV; **E**, **F**) Tumor mutation burden in specific samples. **G** The top 10 mutated genes in SCLC. **H** The prevalence of total and oncogenic alterations in specified signaling pathways in SCLC. SNV: single nucleotide variation

Through targeted deep sequencing of all exons and selected introns of 618 cancer-related genes (Additional file 1: Table S1) in 75 SCLC tissue and blood samples, a total of 97.3% (73/75) of the samples were identified as valid somatic mutations. As shown in Fig. 1A, a total of 978 mutations were identified in 75 cases, with a median mutation number of 13 per patient (range 1–60). Missense mutation was the main mutation type, and the frequency of single nucleotide polymorphism was higher than that of insertion and deletion, C > T was the most common single nucleotide variation in SCLC (Fig. 1B–D). In addition, we counted the number of altered bases in each sample and color-coded the types of mutations (Fig. 1E, F). The top 10 mutated genes with highest prevalence were *TP53* (77.30%), *RB1* (30.70%), *KMT2D* (17.30%), *ALK* (16.00%), *LRP1B* (14.70%), *EGFR* (14.70%), *NOTCH3* (14.70%), *AR* (14.70%), *CREBBP* (12.00%), *ROS1* (12.00%), and *BRCA2* (12.00%), respectively (Fig. 1G). Pathway analysis indicated that the most frequently enriched pathways were Cell Cycle (84.00%), RTK-RAS-MAPK (72.00%), DNA damage repair (DDR, 49.33%), Epigenetic_modifiers/Chromatin_remodelers (40.00%), and NOTCH (36.00%), respectively (Fig. 1H).

### Germline mutations in Chinese SCLC patients

In our cohort, 60.00% (45/75) patients harbored at least one germline mutation, and the total number of germline mutations was 105. The patients with germline mutation were further divided into pathogenic/likely pathogenic and non-pathogenic types, and the patients with pathogenic/likely pathogenic germline mutation accounted for 9.33% of the 75 SCLC patients. These germline mutation genes included *BRCA2, BRCA1, ATM, UCP3, GCDH, MPL, SMO, FGFR4* and *TP53* genes. A deleterious mutation in the germline may indicate family heredity, so we investigated the familial history of cancer in 75 SCLC patients. After excluding 2 patients who were unwilling to provide a family history of cancer, we found that 24.00% (18/75) had a family history of cancer, with most family members being diagnosed with respiratory and digestive tract tumors. We further screened 5 patients with germline mutations and found that only 1 carrier had susceptible genetic hereditary phenomena in his family (Table 2). Notably, this patient has been identified with dual deleterious variants, including an *ATM-c.2376* + *1G* > *A and a TP53-p.Arg273His*.

**Table 2:** Details of pathogenic or likely pathogenic variants carriers

ID	Age	Gender	Family history	Gene	Exon	Nucleotide change	Amino acid change
2,019,772	70	Male	Father, sister	*ATM*	None	c.2376 + 1G > A	
2,019,772	70	Male	Father, sister	*TP53*	8	c.818G > A	p.Arg273His
2,016,599	39	Male	NO	*BRCA1*	10	c.1465G > T	p.Glu489Ter
2,032,699	77	Male	NO	*BRCA1*	15	c.4801A > T	p.Lys1601Ter
2,012,976	68	Male	NO	*BRCA2*	25	c.9294C > G	p.Tyr3098Ter
2,013,902	80	Male	NO	*FGFR4*	4	c.379G > C	p.Asp127His

### Genetic alterations in DNA damage repair pathway

**Fig. 2:**
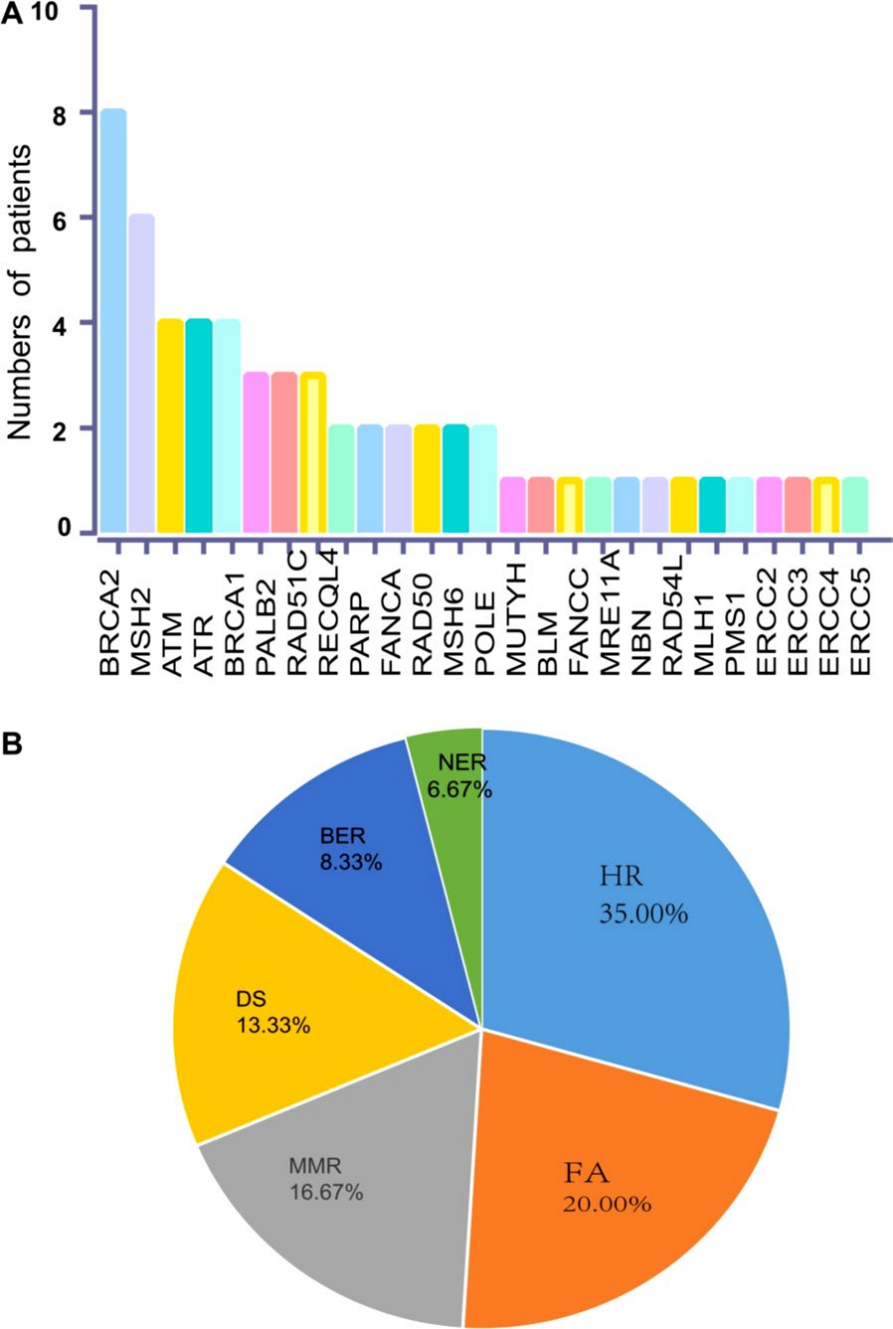
Genetic alterations in DNA damage repair pathway. **A** The distribution of known or likely deleterious somatic DDR gene mutations. **B** Frequency of altered pathway for DDR

A total of 28 patients (37.33%) harbored at least one alteration in DNA repair genes. The distribution of specific genes was exhibited in Fig. 2A, and the most frequently mutated DDR genes with known or likely deleterious variants were *BRCA2* (n = 8, 13.33%) and *MSH2* (n = 6, 10.00%), followed by *ATM* (n = 4, 6.67%), *ATR* (n = 4, 6.67%) and *BRCA1* (n = 4, 6.67%). DDR pathway analysis showed that the most frequently enriched DDR pathways were homology recombination (HR, 35.00%), fanconi anaemia (FA, 20.00%), mismatch repair (MMR, 16.67%), DNA sensor (DS, 13.33%), base excision repair (BER, 8.33%) and nucleotide excision repair (NER, 6.67%) respectively (Fig. 2B). We also analyzed the clinical significance of DDR-related genes and these genes are listed below (Table 3). Particularly, HR pathway accounts for the most among these genes, followed by MMR and DS pathway.

**Table 3:** Altered Genes with Clinical Significance

Gene	OncoKB Annotation	DDR signal pathway	Coding_seq_change
*BRCA2*	Likely Oncogenic	HR	c.9294C > G
*RAD50*	Likely Oncogenic	HR	c.3618delATCTCTTGCCAATGCTCTGGTTGAGTAAGT
*BRCA1*	Likely Oncogenic	HR	c.4801A > T
*BRCA1*	Likely Oncogenic	HR	c.1465G > T
*MSH2*	Likely Oncogenic	MMR	c.640A > T
*ATM*	Likely Oncogenic	DS	c.2376 + 1G > A

### Differences of somatic gene mutations in SCLC patients between our cohort and Western cohorts

**Fig. 3:**
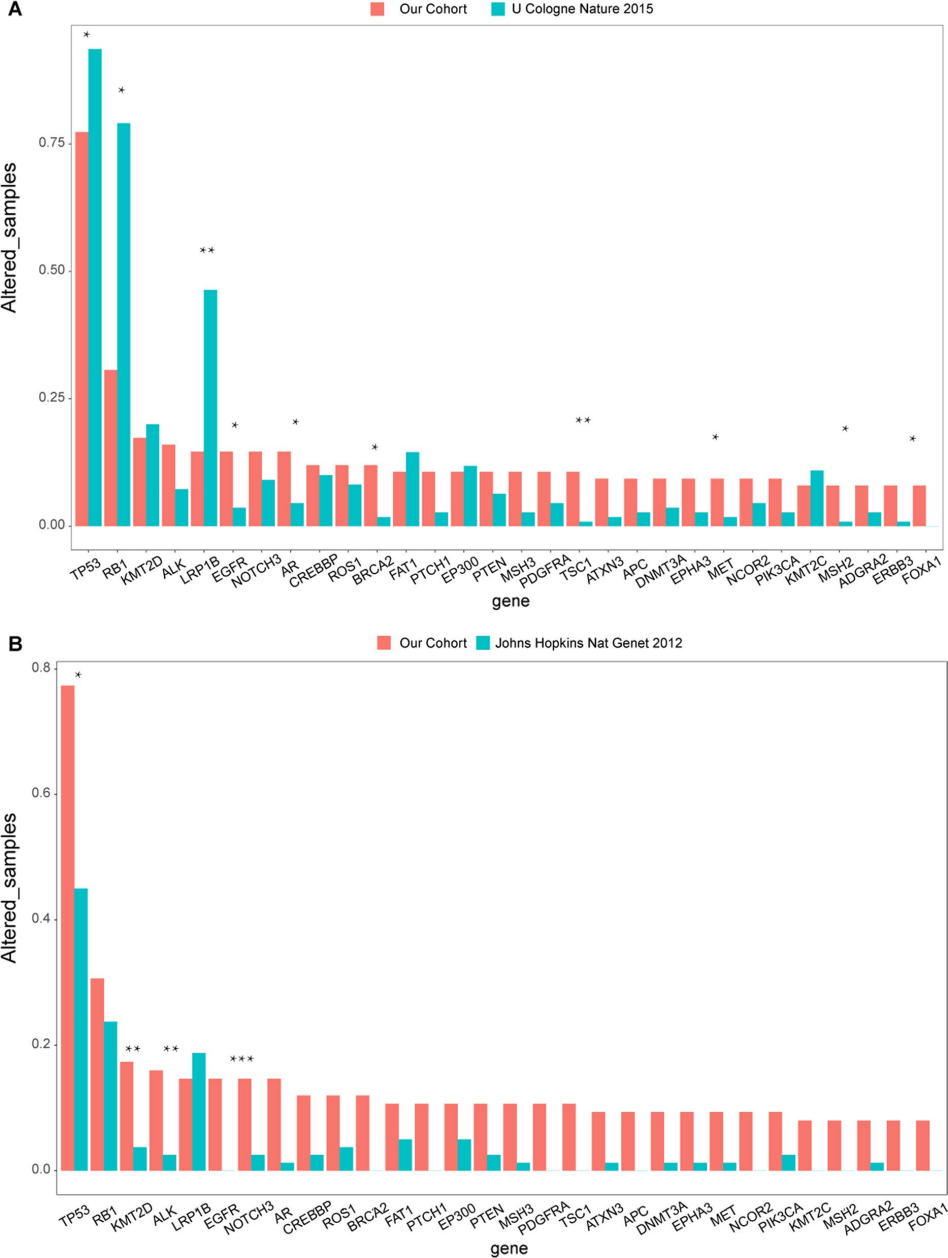
**A** Comparisons of the gene prevalence identified in our cohort (red bars) and U Cologne cohort (green bars). **B** Comparisons of the gene prevalence identified in our cohort and in Johns Hopkins cohort. Two-sided Fisher’s tests were conducted to compare the different frequency between two cohorts. ***p $\le$ 0.001, **p $\le$ 0.01, *p $\le$ 0.05

Comparing the significantly mutated genes with U Cologne cohort showed that there were several significantly lower mutated genes in *RB1* (30.67 vs 79.09%), *LRP1B* (14.67 vs 46.36%) and *TP53* (77.33 vs 93.64%), but a higher prevalence in *EGFR* (14.67% vs 3.64%), *AR* (14.67 vs 4.55%), *BRCA2* (12 vs 1.82%), *TSC1* (10.67 vs 0.91%), *ATXN3* (9.33 vs 1.82%), *MET* (9.33 vs 1.82%), *MSH2* (8 vs 0.91%), *ERBB3* (8 vs 0.91%) and *FOXA1* (8% vs none) were presented in our cohort (Fig. 3A). While compared to the data from Johns Hopkins cohort, a higher mutated frequency in *TP53* (77.33 vs 45.0%), *KMT2D* (17.33 vs 3.75%), *ALK* (16 vs 2.5%), and *EGFR* (14.67% vs none) were found in our cohort (Fig. 3B).

### TMB analysis in the Chinese cohort

**Fig. 4:**
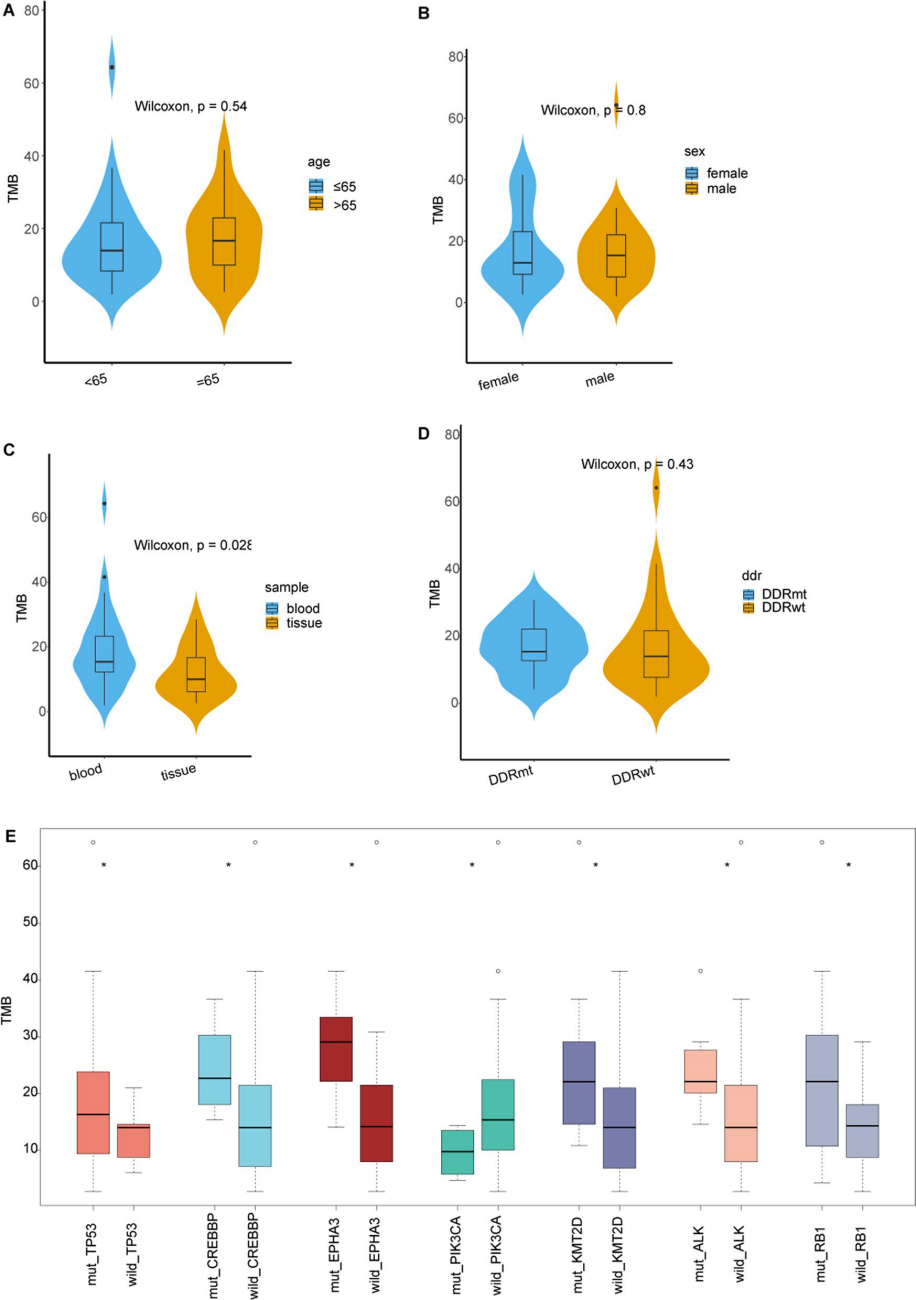
Comparisons of median TMB in Chinese SCLC patients with certain specific gene mutations. DDRmt: DDR mutant; DDRwt: DDR wildtype

The TMB values in our cohort ranged from 2.00/Mb to 64.29/Mb with a median value of 14.53/Mb. And the TMB was significantly higher in blood samples than in the tissue sample group (*p* = 0.028) as more extensive stage cases involved. However, there were no significant differences in TMB were observed between each of these compared groups with age, gender and DDR mutation (Fig. 4A–D). Moreover, the median TMB of patients with alterations in *TP53* (*p* = 0.018), *CREBBP* (*p* = 0.013), *EPHA3* (*p* = 0.013), *KMT2D* (*p* = 0.03), *ALK* (*p* = 0.046) and *RB1* (*p* = 0.05) genes were higher than those without the alterations, on the contrary the median TMB of patients with *PIK3CA* alteration (*p* = 0.019) was lower (Fig. 4E).

### Clinically actionable alterations

**Fig. 5:**
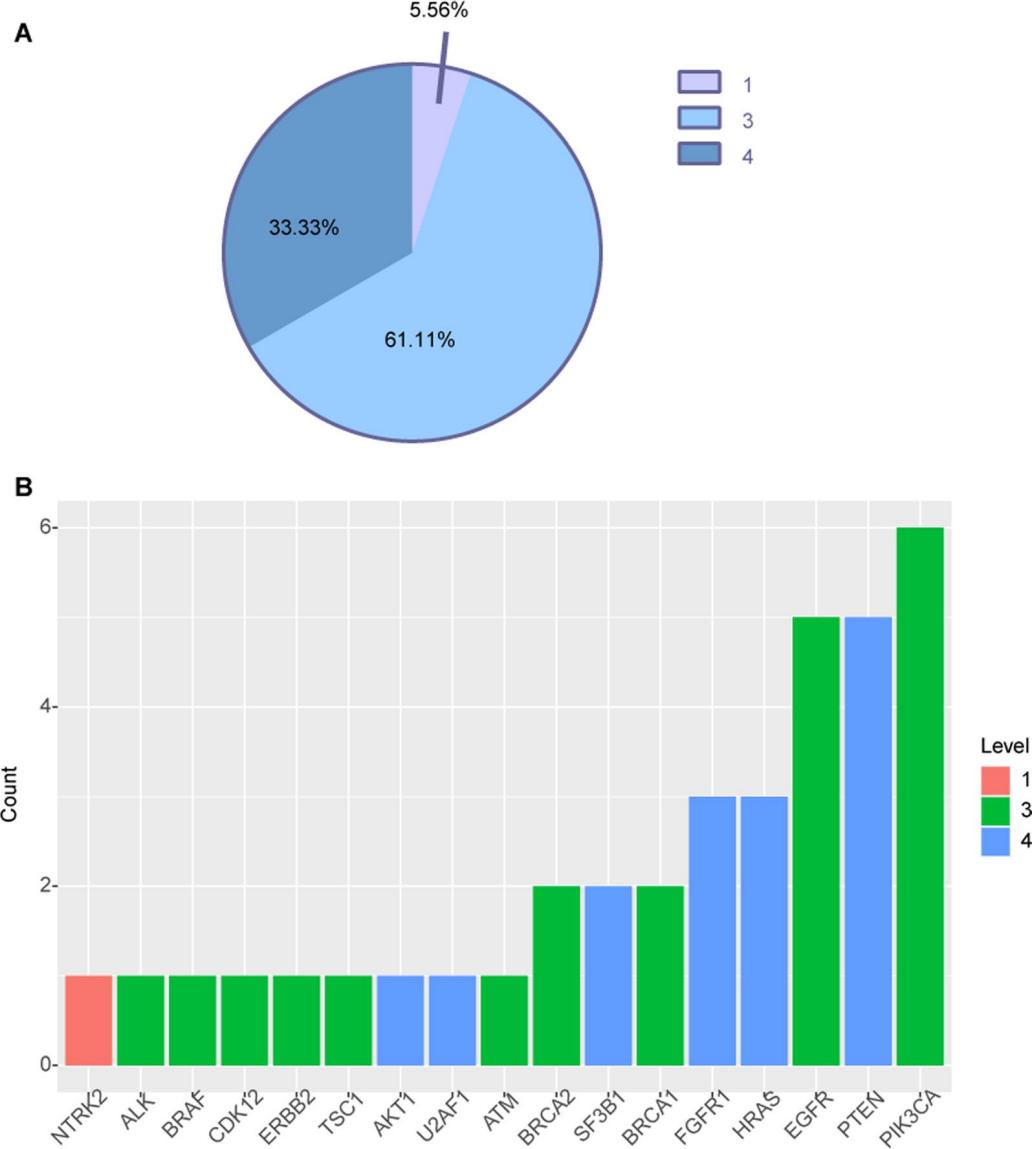
**A** Samples were assigned to the highest level of actionable alterations. **B** Distribution of levels of actionable alterations

To evaluate the clinical utility of prospective molecular profiling to guide treatment decisions, we used OncoKB (http://oncokb.org/) to group all mutations into various levels according to evidence of clinical actionability. Overall, 33.33% (25/75) of patients harbored at least one actionable alteration (Table 4). We found a group of gene mutations as standard care biomarkers for an FDA-approved drug in another indication. 5.56% of tumors harbored level_1 gene alteration (Fig. 5A) including *NTRK* (Fig. 5B). Level_3 accounted for 61.11% (Fig. 5A), including *ALK, BRAF, CDK12, ERBB2, TSC1, ATM, BRCA1/2, EGFR, PIK3CA* (Fig. 5B). Level_4 accounted for 33.33% (Fig. 5A), including *AKT, U2AF1, SF3B1, FGFR1, HRAS* (Fig. 5B). Additionally, two germline alterations including one *BRCA1* germline alterations and one *ATM* germline alteration may confer sensitivity to corresponding target therapy (Table 4) (Fig. 5).

**Table 4:** Actionable Alterations identified in our cohort

Level of evidence based on OncoKB (12/20/2019)	Altered genes	Mutational type	No of patients	(%)	Related drugs
			25	33.33	
1	*NTRK2*	Fusions	1	1.33	Entrectinib, Larotrectinib
3	*ALK*	Fusions	1	1.33	Crizotinib
3	*BRAF*	V600E	1	1.33	Dabrafenib, Dabrafenib + Trametinib, Vemurafenib + Cobimetinib, Encorafenib + Binimetinib, Encorafenib + Cetuximab, Encorafenib + Panitumumab, Trametinib, Vemurafenib
3	*BRCA2*	Oncogenic Mutations	1	1.33	Niraparib, Olaparib, Rucaparib, Rucaparib
3	*CDK12*	Oncogenic Mutations	1	1.33	Olaparib
3	*EGFR*	Exon 19 deletion, T790M	5	6.67	Afatinib, Dacomitinib, Erlotinib, Gefitinib, Osimertinib
3	*ERBB2*	Amplification	1	1.33	Ado-Trastuzumab Emtansine, Capecitabine + Trastuzumab + Tucatinib, Lapatinib + Capecitabine, Lapatinib + Letrozole, Margetuximab + Chemotherapy, Neratinib + Capecitabine, Neratinib, Trastuzumab + Pertuzumab + Chemotherapy, Trastuzumab Deruxtecan, Trastuzumab, Trastuzumab + Chemotherapy, Lapatinib + Trastuzumab, Trastuzumab + Pertuzumab, Carboplatin-Taxol Regimen + Trastuzumab
3	*PIK3CA*	Oncogenic Mutations	6	8.00	Fulvestrant + Alpelisib
3	*TSC1*	Oncogenic Mutations	1	1.33	Everolimus
4	*AKT1*	E17K	1	1.33	AZD5363
4	*FGFR1*	Amplification	3	4.00	Debio1347, BGJ398, Erdafitinib
4	*HRAS*	Oncogenic Mutations	3	4.00	Tipifarnib
4	*PTEN*	Oncogenic Mutations	5	6.67	AZD8186, GSK2636771
4	*SF3B1*	Oncogenic Mutations	2	2.67%	H3B-8800
4	*U2AF1*	Oncogenic Mutations	1	1.33%	H3B-8800
*Other potentially actionable gene*					
3	*BRCA1*	germline	2	4.44%	Niraparib, Olaparib(PARPi)
3	*BRCA2*	germline	1	2.22%	Niraparib,Olaparib(PARPi)
3	*ATM*	germline	1	2.22%	Olaparib (PARPi)

## Discussion

SCLC is an aggressive and refractory form of lung cancer originated from neuroendocrine cells. It must be emphasized that although immune checkpoint therapy has paved the new way for the treatment of SCLC, more precise and effective therapy for SCLC still need to be explored. However, due to the lack of treatable oncogene mutations, molecular targeted therapies for SCLC have not yet been developed. Modern technologies such as next-generation sequencing (NGS) can carry out gene profiling of cancer cells, making the successful development of molecular targeted therapy possible, and has remarkable potential to realize the precision medicine for cancers. In this study, we used NGS to elucidate the genomic characteristics of Chinese SCLC patients, especially DDR alterations and TMB levels, to provide a basis for the development of precision targeted therapy.

As expected, we detected the most frequent mutations in *TP53* (77.3%) and *RB1* (30.7%), which in line with previous publications [6, 22]. In addition to common genomic alterations, alterations in other tumor-related genes displayed a unique feature in Chinese populations. The prevalence of *EGFR, BRCA2, TSC1, KMT2D* and *ALK* gene alterations was higher in the Chinese cohort than in the Western population. Among those differences, *BRCA2* was the well-known biomarkers for PARP inhibitors [23], and TSC1 naturally suppressed the overactivity of downstream mammalian target of rapamycin (mTOR), which indicated the potential clinical benefits of patients with *TSC1* loss of function mutations from mTOR inhibitors [24, 25]. *EGFR* mutation and *ALK* rearrangement are meaningful targetable driver alterations in lung adenocarcinoma (LUAD) and non-small-cell lung cancer (NSCLC), respectively [26, 27]. Histological transformation of *EGFR*-driven or *ALK*-driven LUAD to SCLC has been reported in some cases [28]. The conversion of LUAD to SCLC has been shown to be associated with acquired resistance to EGFR or other tyrosine kinase receptor inhibitors [29–31]. However, the patients enrolled in our study were all patients with primary SCLC who had not been converted to SCLC from other cancer types after multiline therapy. Moreover, the frequency of *EGFR* and *ALK* mutations measured in our cohort is higher than previously reported, which is a finding worthy of further exploration. However, compared with the Western SCLC patients (U Cologne cohort), the incidence of *LRP1B* in Chinese patients with SCLC was lower. Due to its long coding sequence, *LRP1B* is often omitted from genomic research, but its mutation may still have a functional consequence in tumorigenesis and heterogeneity [32]. This gene encoded lipoprotein receptor-related protein 1B, and was suggested as a novel tumor suppressor gene and associated with better efficacy with immunotherapy in NSCLC and melanoma [33]. In patients with multiple primary lung cancers, *LRP1B* alterations were also associated with higher TMB value and positive tumor PDL1 expression [34]. There was poor number of studies reporting on the prevalence and function of *LRP1B* in SCLC, and our study provided a clue of the difference role of it in the carcinogenesis of SCLC between Western and Chinese patients. Whether loss of function or deletion of *LRP1B* related to the clinical outcome of *LRP1B* inhibitors was not clear, but the lower incidence of this gene may indicate the differences in the pathogenesis between different ethnic groups. Our genomic analyses further compared the genetic alterations involved in several cancer-related signaling pathways in the Chinese cohort. We found that most of the mutant genes were enriched in the Cell Cycle, RTK-RAS-MAPK and DDR signaling pathways, suggesting that the molecular characterization of these pathways is closely related to the development of SCLC.

Previous studies have similarly examined the prevalence and spectrum of germline variants in SCLC patients, but they are primarily focused on limited genes or in a small subset [35]. Our findings provide a novel insight on the SCLC with germline alterations in the Chinese population tested by an NGS panel with 618 cancer-related genes. Specifically, 9.33% of Chinese SCLC patients had pathogenic or likely pathogenic germline gene variants, including *BRCA2, BRCA1, ATM, UCP3, GCDH, MPL, SMO, FGFR4* and *TP53*. Moreover, 24% of patients had a family history of cancer, highlighting the necessity of risk assessment for those patients and their first-degree family members. Additionally, some publications have investigated the roles of germline alternations, mostly selected mutations, in genetic susceptibility to lung cancer [36, 37], while systematic studies of the germline mutations potentially predisposing to lung cancer. For example, the identification of germline mutations in driver oncogenes like *EGFR*, has heightened interest in identifying germline mutations carrying a high inherited risk of lung cancer [38]. However, *EGFR* mutations are not conventional germline mutations associated with hereditary cancers, and are not common in our cohort as well [39]. Liu et al. found that *BRCA2* and *ATM* were germline mutations with the highest mutation frequency in Chinese lung cancer patients, similar to our results [40].

Unlike NSCLC, SCLC harbors few actionable mutations that can be used for therapeutic intervention. Actionability is defined as a molecular alteration that has clinical or strong preclinical evidence of a predictive benefit from a specific therapy (in any cancer type) [41, 42]. Here, we detected that 33.33% of SCLC patients had at least one actionable alteration with any level of evidence from OncoKB. Our results provide a new insight into patients with SCLC tumors who harbor actionable molecular alterations and receive appropriately matched therapy. Pishvaian’s investigation showed that patients with actionable molecular alterations could benefit considerably from receiving matched therapy [43]. It has been reported that patients with advanced pancreatic cancer with actionable alterations who received matched therapy had a one-year increase in median overall survival compared with patients with or without actionable alterations who did not receive matched therapy. However, other therapeutic modality did not offer such a huge advantage for this patient population. Thus, these findings set the stage for prospective clinical trials guided by molecular profiling. Previous findings revealed that the median PFS of patients with actionable alterations undergoing molecularly matched therapies is significantly longer than that of historical controls. To our knowledge, there is no systematic assessment of median overall survival of SCLC patients with molecularly matched therapies [44]. The sensitivity of these analyses to molecular profiling warrants further investigation.

DDR pathway defects may lead to severe DNA damage, resulting in genome instability and trigger malignant transformation [45]. Therefore, targeting the DDR pathway may be a promising therapeutic strategy for SCLC [9, 46]. The high frequency of DDR gene and pathway alterations in our cohort and other studies identifies opportunities to improve cancer therapy. For example, HR defects are relatively common in cancer and may compromise DNA replication and genome stability [47]. Thus, combination therapies that induce or potentiate replication stress or impair replication fork protection may effectively inhibit HR-deficient cancers like SCLC. PARP inhibitors have demonstrated great promise in the treatment of patients with deficiencies in HR DNA repair. Among the DDR proteins, PARP inhibitors are the most attractive agents in clinical research [48]. Farago et al. conducted a phase I/II trial combining olaparib (PARP inhibitor) with temozolomide in previously treated SCLC patients. The results showed that the overall response rate was 41.7%, the median overall survival was 8.5 months, and the median progression-free survival was 4.2 months [49]. Their findings provide a promising new therapeutic strategy for SCLC. PARP inhibitors are active in SCLC models and clinical trials are in progress as well [9, 13], so the clinical benefit of these biomarker-targeted therapies for patients with SCLC will hopefully be realized.

This study also has some limitations. Firstly, serial analyses of tumor biopsies have not been performed in some SCLC patients, limiting molecular studies and biomarker assessments of treatment-induced changes in this cancer type. Secondly, due to the limited sample size, the results may have some deviation.

## Conclusions

Our study describes the clinical characteristics of SCLC in China and identifies many novel candidate genes, some of which may have therapeutic implications. Our results further figure out there were significant differences between our cohort and other two cohorts from cBioportal database of the mutational data. Analysis of these altered genes provided information regarding the molecular mechanisms of SCLC and significant biomarkers or targets for the diagnosis and treatment of SCLC. However, further molecular biological experiments are required to confirm the function of the pathways in SCLC.

## Supplementary Information

**Additional file 1: Table S1.** 618 selected cancer-related genes.

## Data Availability

The datasets used and/or analyzed during the current study are available from the corresponding author on reasonable request.

## Abbreviations

SCLC: Small cell lung cancer
DDR: DNA damage repair
TMB: Tumor mutation burden
FA: Fanconi anaemia
HR: Homology recombination
MMR: Mismatch repair
DS: DNA sensor
BER: Base excision repair
NER: Nucleotide excision repair
OS: Overall survival
ICI: Immune checkpoint inhibitors
PARP: Poly ADP-ribose polymerase
FFPE: Formalin-fixed, paraffin-embedded
cfDNA: Cell-free DNA
