# Supplementary material for: Genomic features of Chinese small cell lung cancer
Source: BMC Med Genomics. 2022 May 20;15:117. doi: 10.1186/s12920-022-01255-3 (PMC9123817; doi:10.1186/s12920-022-01255-3)
Supplement: Supplementary file 1 — Additional file 1: Table S1. 618 selected cancer-related genes. [file 12920_2022_1255_MOESM1_ESM.docx]

Supplementary Table1 618 selected cancer-related genes

| **Gene** | **Pathway** |
| --- | --- |
| *ABL1* | RAS/RAF/MAPK |
| *ABL2* | Other |
| *ACVR1B* | TGF-Beta |
| *ADGRA2* | Other |
| *AGO2* | Translational Control |
| *AKT1* | PI3K |
| *AKT2* | PI3K |
| *ALK* | RAS/RAF/MAPK |
| *AMER1* | WNT/CTNNB1 |
| *ANKRD11* | Other |
| *APC* | WNT/CTNNB1 |
| *AR* | Other |
| *ARAF* | RAS/RAF/MAPK |
| *ARFRP1* | Other |
| *ARID1A* | Epigenetic_modifiers/Chromatin_remodelers |
| *ARID1B* | Epigenetic_modifiers/Chromatin_remodelers |
| *ARID2* | Epigenetic_modifiers/Chromatin_remodelers |
| *ASXL1* | Other |
| *ASXL2* | Other |
| *ATM* | DNA repair |
| *ATR* | DNA repair |
| *ATRX* | DNA repair |
| *AURKA* | Cell cycle |
| *AURKB* | Cell Cycle |
| *AXIN1* | WNT/CTNNB1 |
| *AXIN2* | Wnt/Hedgehog/Notch |
| *AXL* | RAS/RAF/MAPK |
| *BABAM1* | DNA repair |
| *BAP1* | Other |
| *BARD1* | DNA repair |
| *BCL10* | NF-kappaB Signaling |
| *BCL11A* | Other |
| *BCL2* | Apoptosis and Autophagy |
| *BCL2L1* | Other |
| *BCL2L11* | Apoptosis and Autophagy |
| *BCL6* | Other |
| *BCOR* | DNA repair |
| *BCORL1* | Other |
| *BIRC3* | Apoptosis and Autophagy |
| *BIRC5* | Other |
| *BLM* | DNA repair |
| *BMPR1A* | Other |
| *BRAF* | RAS/RAF/MAPK |
| *BRCA1* | DNA repair |
| *BRCA2* | DNA repair |
| *BRD3* | Other |
| *BRD4* | Chromatin Regulation/Acetylation |
| *BRIP1* | DNA repair |
| *BUB1B* | DNA repair |
| *CALR* | Other |
| *CARD11* | Other |
| *CASP8* | Apoptosis and Autophagy |
| *CBFB* | Other |
| *CBL* | NF-kappaB Signaling |
| *CCND1* | Cell cycle |
| *CCND2* | Cell Cycle |
| *CCND3* | Cell Cycle |
| *CCNE1* | Cell cycle |
| *CD276* | NF-kappaB Signaling |
| *CD74* | NF-kappaB Signaling |
| *CD79B* | Other |
| *CDC73* | Wnt/Hedgehog/Notch |
| *CDH1* | EMT |
| *CDK12* | DNA repair |
| *CDK4* | Cell cycle |
| *CDK6* | Cell cycle |
| *CDK8* | Cell cycle |
| *CDKN1A* | Cell Cycle |
| *CDKN2A* | Cell cycle |
| *CEBPA* | NF-kappaB Signaling |
| *CHD2* | Chromatin Regulation/Acetylation |
| *CHD4* | Chromatin Regulation/Acetylation |
| *CHEK2* | DNA repair |
| *CIC* | Other |
| *CREBBP* | NOTCH |
| *CSF1R* | ERK |
| *CSF3R* | RAS/RAF/MAPK |
| *CTCF* | Chromatin Regulation/Acetylation |
| *CTNNB1* | WNT/CTNNB1 |
| *CUL3* | DNA repair |
| *CYSLTR2* | Other |
| *DAXX* | Other |
| *DDB2* | DNA repair |
| *DDR2* | Tyrosine Kinases/Adaptors |
| *DICER1* | Other |
| *DNMT1* | Epigenetic_modifiers/Chromatin_remodelers |
| *DNMT3A* | Epigenetic_modifiers/Chromatin_remodelers |
| *DNMT3B* | Chromatin Regulation/Acetylation |
| *DOT1L* | Other |
| *DROSHA* | Translational Control |
| *E2F1* | Cell cycle |
| *E2F3* | Cell Cycle |
| *EED* | Other |
| *EGF* | RAS/RAF/MAPK |
| *EGFR* | RAS/RAF/MAPK |
| *ELF3* | Other |
| *EP300* | NOTCH |
| *EPAS1* | Other |
| *EPCAM* | DNA repair |
| *EPHA3* | Other |
| *EPHA5* | Other |
| *EPHA7* | Other |
| *EPHB1* | Angiogenesis |
| *ERBB2* | RAS/RAF/MAPK |
| *ERBB3* | RAS/RAF/MAPK |
| *ERBB4* | RAS/RAF/MAPK |
| *ERCC2* | DNA repair |
| *ERCC3* | DNA repair |
| *ERCC4* | DNA repair |
| *ERCC5* | DNA repair |
| *ERF* | RAS/RAF/MAPK |
| *ERG* | Chromatin Regulation/Acetylation |
| *ERRFI1* | RAS/RAF/MAPK |
| *ESR1* | Other |
| *ETV1* | RAS/RAF/MAPK |
| *ETV5* | Other |
| *ETV6* | Other |
| *EWSR1* | Chromatin Regulation/Acetylation |
| *EXT2* | Other |
| *EZH2* | Chromatin Regulation/Acetylation |
| *FAM175A* | DNA repair |
| *FAM46C* | Other |
| *FANCA* | DNA repair |
| *FANCC* | DNA repair |
| *FANCF* | DNA repair |
| *FANCG* | DNA repair |
| *FANCM* | DNA repair |
| *FAS* | NF-kappaB Signaling |
| *FAT1* | HIPPO |
| *FAT4* | HIPPO |
| *FBXW7* | PI3K |
| *FGF10* | MAPK |
| *FGF19* | MAPK |
| *FGF3* | MAPK |
| *FGF4* | RAS/RAF/MAPK |
| *FGF6* | RAS/RAF/MAPK |
| *FGFR1* | RAS/RAF/MAPK |
| *FGFR2* | RAS/RAF/MAPK |
| *FGFR3* | RAS/RAF/MAPK |
| *FGFR4* | RAS/RAF/MAPK |
| *FH* | Glucose / Energy Metabolism |
| *FLT1* | RAS/RAF/MAPK |
| *FLT3* | RAS/RAF/MAPK |
| *FLT4* | RAS/RAF/MAPK |
| *FOLR3* | Other |
| *FOXA1* | Other |
| *FOXL2* | Other |
| *FOXP1* | Other |
| *FUBP1* | Other |
| *FYN* | RAS/RAF/MAPK |
| *GABRA6* | Other |
| *GATA2* | NF-kappaB Signaling |
| *GATA3* | NF-kappaB Signaling |
| *GATA4* | Other |
| *GATA6* | Other |
| *GEN1* | DNA repair |
| *GID4* | Other |
| *GLI1* | Hedgedog |
| *GLI2* | Hedgedog |
| *GNA11* | RAS/RAF/MAPK |
| *GNA13* | Other |
| *GNAQ* | RAS/RAF/MAPK |
| *GNAS* | RAS/RAF/MAPK |
| *GOPC* | Cytoskeletal Signaling |
| *GPC3* | Wnt |
| *GPS2* | RAS/RAF/MAPK |
| *GREM1* | Wnt/Hedgehog/Notch |
| *GRIN2A* | PI3K |
| *GRM3* | Other |
| *H3F3A* | Other |
| *HDAC1* | Epigenetic_modifiers/Chromatin_remodelers |
| *HDAC3* | Chromatin Regulation/Acetylation |
| *HDAC4* | Chromatin Regulation/Acetylation |
| *HDAC6* | Epigenetic_modifiers/Chromatin_remodelers |
| *HGF* | Angiogenesis |
| *HIST1H1C* | Other |
| *HIST1H3B* | Other |
| *HIST1H3F* | Other |
| *HIST1H3J* | Cell Cycle |
| *HIST3H3* | MAPK |
| *HNF1A* | PI3K |
| *HOXB13* | Other |
| *HRAS* | RAS/RAF/MAPK |
| *IDH1* | DNA repair |
| *IDH2* | Glucose / Energy Metabolism |
| *IFNGR1* | Other |
| *IGF1* | ERK |
| *IGF1R* | RAS/RAF/MAPK |
| *IGF2* | ERK |
| *IKBKE* | NF-kappaB Signaling |
| *IKZF1* | Other |
| *IL10* | NF-kappaB Signaling |
| *IL7R* | MAPK |
| *INHBA* | Other |
| *INPP4B* | PI3K/Akt Signaling |
| *INPPL1* | PI3K |
| *IRS1* | Glucose/Energy Metabolism |
| *IRS2* | RAS/RAF/MAPK |
| *JAK2* | RAS/RAF/MAPK |
| *JAK3* | RAS/RAF/MAPK |
| *JUN* | RAS/RAF/MAPK |
| *KAT6A* | Other |
| *KDM5A* | Chromatin Regulation/Acetylation |
| *KDM5C* | Epigenetic_modifiers/Chromatin_remodelers |
| *KDM6A* | Chromatin Regulation/Acetylation |
| *KDR* | RAS/RAF/MAPK |
| *KEAP1* | NRF |
| *KEL* | Other |
| *KIT* | RAS/RAF/MAPK |
| *KLF4* | Wnt/Hedgehog/Notch |
| *KLHL6* | Other |
| *KMT2A* | Epigenetic_modifiers/Chromatin_remodelers |
| *KMT2B* | Epigenetic_modifiers/Chromatin_remodelers |
| *KMT2C* | Epigenetic_modifiers/Chromatin_remodelers |
| *KMT2D* | Epigenetic_modifiers/Chromatin_remodelers |
| *KNSTRN* | Other |
| *KRAS* | RAS/RAF/MAPK |
| *LATS1* | HIPPO |
| *LATS2* | DNA repair |
| *LRP1B* | Other |
| *LRRK2* | RAS/RAF/MAPK |
| *LYN* | RAS/RAF/MAPK |
| *MAGI2* | RAS/RAF/MAPK |
| *MALT1* | NF-kappaB Signaling |
| *MAP2K1* | RAS/RAF/MAPK |
| *MAP2K4* | MAPK |
| *MAP3K1* | RAS/RAF/MAPK |
| *MAP3K13* | MAPK |
| *MAP3K14* | NF-kappaB Signaling |
| *MAPK1* | RAS/RAF/MAPK |
| *MAPK3* | MAPK |
| *MCL1* | Apoptosis and Autophagy |
| *MDM2* | Cell cycle |
| *MED12* | Cell cycle |
| *MEN1* | Other |
| *MET* | RAS/RAF/MAPK |
| *MGA* | Other |
| *MITF* | Other |
| *MLH1* | DNA repair |
| *MPL* | RAS/RAF/MAPK |
| *MRE11A* | DNA repair |
| *MSH2* | DNA repair |
| *MSH3* | DNA repair |
| *MSH6* | DNA repair |
| *MTOR* | PI3K |
| *MUTYH* | DNA repair |
| *MYC* | MYC |
| *MYCL* | MYC |
| *MYCN* | MYC |
| *MYD88* | NF-kappaB Signaling |
| *NBN* | DNA repair |
| *NCOA3* | Chromatin Regulation/Acetylation |
| *NCOR1* | Other |
| *NF1* | RAS/RAF/MAPK |
| *NF2* | RAS/RAF/MAPK |
| *NFE2L2* | NRF |
| *NFKBIA* | NF-kappaB Signaling |
| *NKX2-1* | Other |
| *NOTCH1* | NOTCH |
| *NOTCH2* | NOTCH |
| *NOTCH3* | NOTCH |
| *NOTCH4* | NOTCH |
| *NRAS* | RAS/RAF/MAPK |
| *NRG1* | RAS/RAF/MAPK |
| *NSD1* | Other |
| *NTRK1* | RAS/RAF/MAPK |
| *NTRK3* | RAS/RAF/MAPK |
| *NUP93* | Other |
| *OPRM1* | Other |
| *PAK3* | Cytoskeletal Signaling |
| *PALB2* | DNA repair |
| *PARK2* | Other |
| *PARP1* | DNA repair |
| *PARP2* | DNA repair |
| *PBRM1* | Epigenetic_modifiers/Chromatin_remodelers |
| *PDCD1* | Other |
| *PDCD1LG2* | Other |
| *PDGFRA* | RAS/RAF/MAPK |
| *PDGFRB* | RAS/RAF/MAPK |
| *PDK1* | Glucose/Energy Metabolism |
| *PDPK1* | PI3K |
| *PEG3* | Other |
| *PGR* | Other |
| *PHOX2B* | Other |
| *PIK3C2B* | PI3K |
| *PIK3C2G* | PI3K |
| *PIK3C3* | PI3K |
| *PIK3CA* | PI3K |
| *PIK3CB* | PI3K |
| *PIK3CD* | PI3K/Akt Signaling |
| *PIK3CG* | PI3K |
| *PIK3R1* | PI3K |
| *PIK3R2* | PI3K/Akt Signaling |
| *PIM1* | MYC |
| *PLCG2* | Other |
| *PLK1* | DNA repair |
| *PMS1* | DNA repair |
| *PMS2* | DNA repair |
| *POLD1* | DNA repair |
| *POLE* | DNA repair |
| *PPARG* | Glucose/Energy Metabolism |
| *PPM1D* | DNA repair |
| *PPP2R1A* | PI3K |
| *PRDM1* | NF-kappaB Signaling |
| *PREX2* | PI3K |
| *PRKAR1A* | Other |
| *PRKCE* | Ca, cAMP and Lipid Signaling |
| *PRKCI* | Other |
| *PRKD1* | Other |
| *PRKDC* | DNA repair |
| *PTCH1* | Hedgedog |
| *PTCH2* | Hedgedog |
| *PTEN* | PI3K |
| *PTK2* | Tyrosine Kinases/Adaptors |
| *PTPN11* | RAS/RAF/MAPK |
| *PTPRD* | Other |
| *PTPRS* | EMT |
| *PTPRT* | Other |
| *RAB35* | Cytoskeletal Signaling |
| *RAD50* | DNA repair |
| *RAD51B* | DNA repair |
| *RAD51D* | DNA repair |
| *RAD52* | DNA repair |
| *RAD54L* | DNA repair |
| *RAF1* | RAS/RAF/MAPK |
| *RARB* | Other |
| *RASA1* | MAPK |
| *RASSF1* | RAS/RAF/MAPK |
| *RB1* | Cell cycle |
| *RBM10* | Other |
| *RECQL* | DNA repair |
| *RECQL4* | DNA repair |
| *RET* | RAS/RAF/MAPK |
| *RHEB* | PI3K |
| *RHOA* | Other |
| *RICTOR* | PI3K |
| *RIT1* | RAS/RAF/MAPK |
| *RNF43* | Wnt |
| *ROCK1* | Other |
| *ROS1* | RAS/RAF/MAPK |
| *RPS6KB1* | PI3K |
| *RPS6KB2* | PI3K |
| *RPTOR* | PI3K |
| *RTEL1* | DNA repair |
| *RUNX1* | NF-kappaB Signaling |
| *RUNX1T1* | Other |
| *RUNX2* | Other |
| *SDHA* | HIF |
| *SDHC* | Other |
| *SESN1* | PI3K |
| *SETD2* | Epigenetic_modifiers/Chromatin_remodelers |
| *SF3B1* | Splicing |
| *SH2B3* | Other |
| *SHOC2* | MAPK |
| *SLIT2* | Other |
| *SLX4* | DNA repair |
| *SMAD3* | TGF-Beta |
| *SMAD4* | TGF-Beta |
| *SMARCA1* | Epigenetic_modifiers/Chromatin_remodelers |
| *SMARCA4* | Epigenetic_modifiers/Chromatin_remodelers |
| *SMARCB1* | Chromatin Regulation/Acetylation |
| *SMO* | Hedgedog |
| *SNAI1* | WNT/CTNNB1 |
| *SNCAIP* | Other |
| *SOS1* | MAPK |
| *SOX10* | Other |
| *SOX17* | Other |
| *SOX2* | Other |
| *SOX9* | Other |
| *SPEN* | Other |
| *SPOP* | NOTCH |
| *SPTA1* | Other |
| *SRC* | MAP Kinase Signaling/Tyrosine Kinases / Adaptors |
| *STAG2* | Chromatin Regulation/Acetylation |
| *STAT4* | NF-kappaB Signaling |
| *STAT5B* | NF-kappaB Signaling |
| *STK11* | PI3K |
| *SYK* | Other |
| *TAF1* | Chromatin Regulation/Acetylation |
| *TBX3* | Other |
| *TCF3* | NF-kappaB Signaling |
| *TCF7L2* | Wnt/Hedgehog/Notch |
| *TEK* | Tyrosine Kinases/Adaptors/Angiogenesis |
| *TERT* | Other |
| *TET1* | Other |
| *TET2* | Chromatin Regulation/Acetylation |
| *TGFBR1* | TGF-Beta |
| *TGFBR2* | TGF-Beta |
| *TMPRSS2* | Other |
| *TNFAIP3* | NF-kappaB Signaling |
| *TOP2A* | Chromatin Regulation/Acetylation |
| *TP53* | cell cycle |
| *TP53BP1* | DNA repair |
| *TP63* | Cell cycle |
| *TRAF2* | NF-kappaB Signaling |
| *TSC1* | PI3K |
| *TSC2* | PI3K |
| *TSHR* | Other |
| *U2AF1* | Translational Control |
| *VHL* | RAS/RAF/MAPK |
| *WHSC1L1* | Other |
| *WISP3* | WNT/CTNNB1 |
| *WNT1* | WNT/CTNNB1 |
| *WNT5A* | Wnt/Hedgehog/Notch |
| *XIAP* | Apoptosis and Autophagy |
| *XPO1* | Chromatin Regulation/Acetylation |
| *YAP1* | Wnt/Hedgehog/Notch |
| *YES1* | MAPK |
| *ZFHX3* | MYC |
| *ZNF217* | Other |
| *ZNF703* | Other |
